# Two Health-Seekers in California

**Published:** 1897-05

**Authors:** 


TWO HEALTH-SEEKERS IN CALIFORNIA. By WILLIAM A. EDWARDS, M. D., Fellow of the College of Physicians of Philadelphia, etc., etc., and BEATRICE HARRADEN, Author of Ships that Pass in the Night, etc. Philadelphia: J. B. Lippincott & Co. 1897.

Dr. Edwards has lived eight years in Southern California and understands the climate thoroughly. Those who take him as their guide in health-seeking will be spared disappointments which must come to many who go with but meager knowledge of the country. He also does a kindness to those of limited means in warning them against going there. Miss Harraden contributes two chapters, gracefully written, but betraying more disappointment in the climate than a native of the northeastern section of the United States, unfamiliar with the tender charms of a more caressing land, will be likely to feel. The book contains many things which a would-be health-seeker should know. M. J. F.

